# Supplementary material for: Associations between Hunger and Psychological Outcomes: A Large-Scale Ecological Momentary Assessment Study
Source: Nutrients. 2022 Dec 5;14(23):5167. doi: 10.3390/nu14235167 (PMC9736756; doi:10.3390/nu14235167)
Supplement: Supplementary file 1 [file nutrients-14-05167-s001.zip › Nutrients_supplementary_figures.pdf]

Supplementary figures

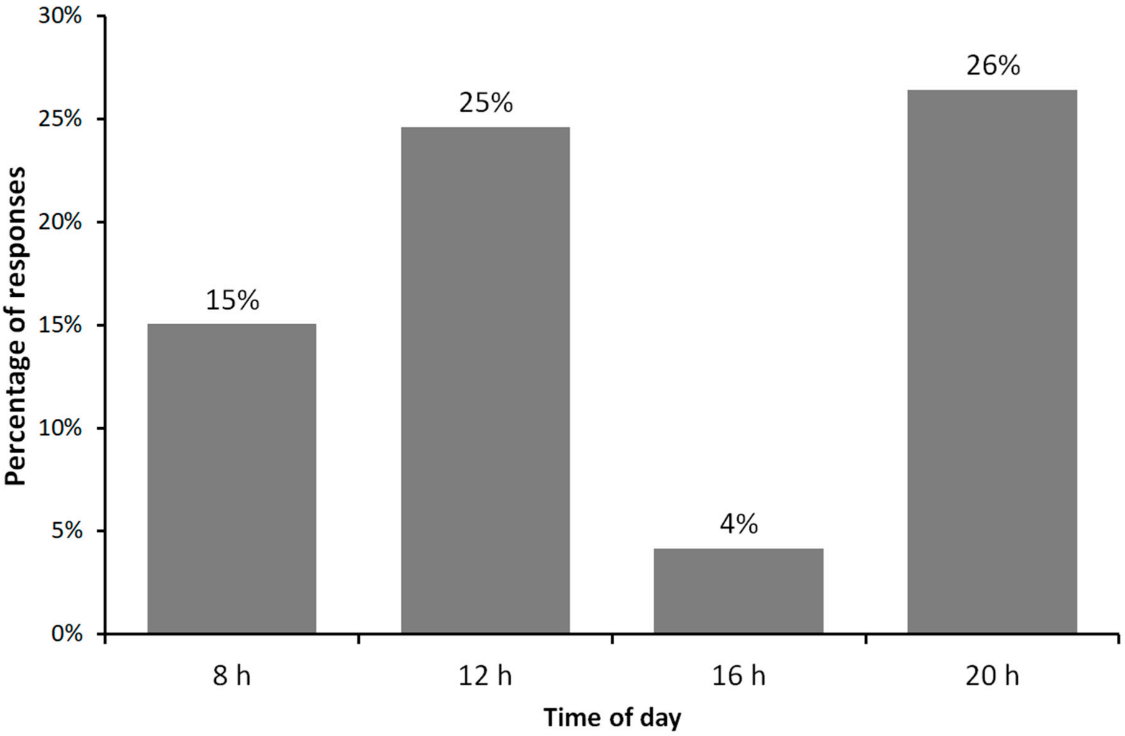

Figure S1: distribution of the responses currently eating according to day period, CoLaus|PsyCoLaus study, Lausanne, Switzerland, 2015-2017.

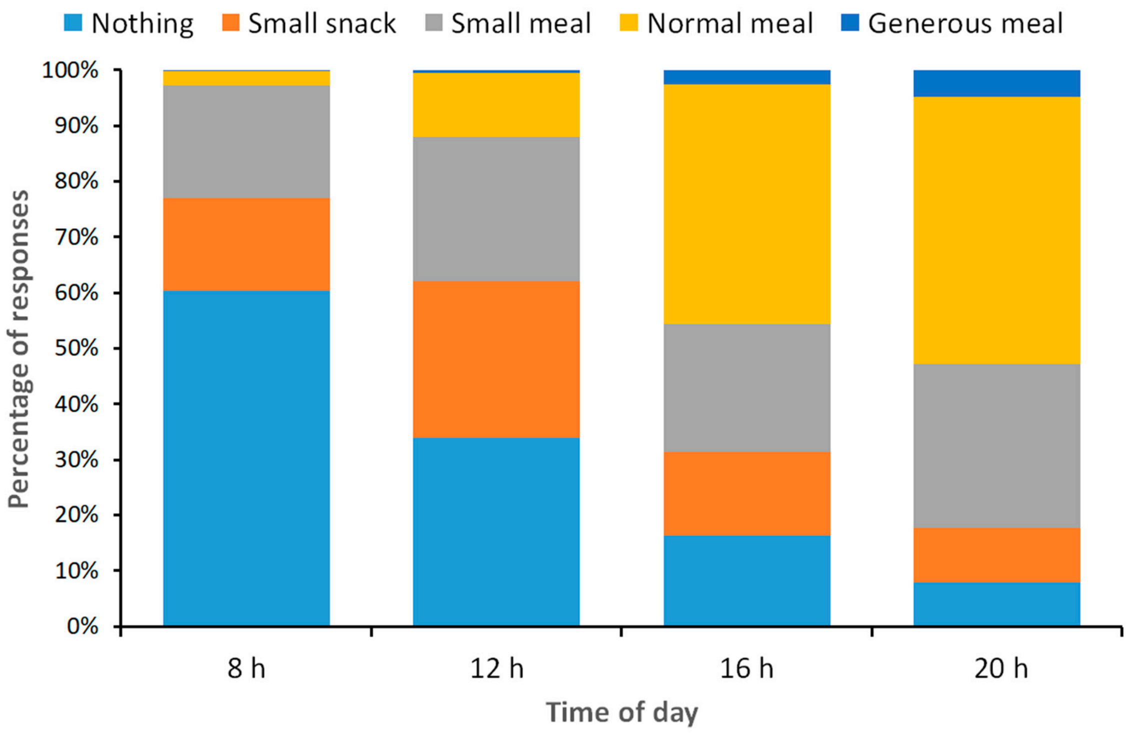

Figure S2: distribution of the responses regarding the type of meal consumed previously according to day period, CoLaus|PsyCoLaus study, Lausanne, Switzerland, 2015-2017.
