# Supplementary material for: Associations between Hunger and Psychological Outcomes: A Large-Scale Ecological Momentary Assessment Study
Source: Nutrients. 2022 Dec 5;14(23):5167. doi: 10.3390/nu14235167 (PMC9736756; doi:10.3390/nu14235167)
Supplement: Supplementary file 1 [file nutrients-14-05167-s001.zip › Nutrients_Table S1_R1_clean.pdf]

Supplementary Tables

Table S1: levels of hunger and psychological states according to day period, CoLaus|PsyCoLaus study, Lausanne, Switzerland, 2015-2017.

	**08:00**	**12:00**	**16:00**	**20:00**	**P-value**
Hunger	2.37 ± 0.03	3.48 ± 0.03	1.70 ± 0.03	1.83 ± 0.03	<0.001
Sadness	2.68 ± 0.03	2.34 ± 0.03	2.39 ± 0.03	2.28 ± 0.03	<0.001
Anxiety	2.12 ± 0.03	2.08 ± 0.03	2.09 ± 0.03	1.91 ± 0.03	<0.001
Feeling active	3.27 ± 0.04	4.11 ± 0.04	4.06 ± 0.04	3.12 ± 0.04	<0.001
Feeling lively	4.23 ± 0.03	4.64 ± 0.03	4.46 ± 0.03	4.07 ± 0.03	<0.001
Feeling distracted	3.08 ± 0.03	2.69 ± 0.03	2.73 ± 0.03	3.12 ± 0.03	<0.001
Anhedonia	1.64 ± 0.03	1.66 ± 0.03	1.67 ± 0.03	1.58 ± 0.03	<0.001
Anger	3.10 ± 0.03	2.57 ± 0.03	2.66 ± 0.03	2.99 ± 0.03	<0.001
Slow thinking	2.81 ± 0.03	2.40 ± 0.03	2.42 ± 0.03	2.33 ± 0.03	<0.001
Restlessness	1.86 ± 0.03	2.00 ± 0.03	1.95 ± 0.03	1.80 ± 0.03	<0.001

Results are expressed as adjusted average ± standard error. Between-period comparisons performed using a mixed model taking into account repeated measures for each participant.
